# Development and validation of a HPLC-MS/MS method with electrospray ionization for quantitation of potassium oxonate in human plasma: Application to a pharmacokinetic study

**DOI:** 10.3892/etm.2013.908

**Published:** 2013-01-18

**Authors:** GUANGTAO HAO, SHAOBO BAI, HAIXIA LIANG, YUGUANG LIANG, HENGYAN QU, HONGZHI GAO, YUANYUAN LI, ZHUANJIE ZHENG, XIAOFANG WANG, ZEYUAN LIU

**Affiliations:** 1 Department of Pharmacology, Affiliated Hospital of the Academy of Military Medical Sciences, Beijing 100071;; 2 Pharmacy Department, The Fifth Hospital of PLA, Yinchuan 750004;; 3 Department of Pharmacy, Beijing Anding Hospital, Capital Medical University, Beijing 100088, P.R. China

**Keywords:** potassium oxonate, HPLC-MS/MS, pharmacokinetics

## Abstract

A rapid, sensitive and specific HPLC-MS/MS method was developed and validated for the quantification of potassium oxonate (Oxo) in human plasma using [^13^C_2_,^15^N_3_]-Oxo as an internal standard (IS). The target substance was separated from human plasma using the solid-phase extraction method. Chromatography separation was performed on a Waters:Atlantis dC^18^ column (150×4.6 mm, 5.0 *μ*m) with a mobile phase consisting of H_2_O with 0.1% formic acid in acetonitrile (90:10, v/v). The mass spectrometer works with electrospray ionization and multiple reaction monitoring in its negative ion mode, using target ions at [M–H]^−^ m/z 111.9 for Oxo and [M–H]^−^ m/z 117.0 for the IS. The mean standard curve was linear (r=0.9991) over the concentration range of 2.0–200.0 ng/ml and had good back-calculated accuracy and precision. The intra- and inter-day precision were <6.33% and the accuracy was >99.38%. The extraction recovery was >60.26%. The lower limit of quantification achieved with this method was 2.0 ng/ml. This assay method was demonstrated to be accurate, sensitive and simple and was successfully applied to a pharmacokinetic study following single oral administration of a 40-mg S-1 capsule in 12 tumor patients.

## Introduction

**Figure 1.**
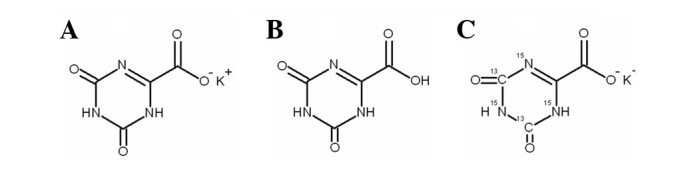
Chemical structures of (A) Oxo, (B) oxonic acid and (C) [^13^C_2_,^15^N_3_]-Oxo. Oxo, potassium oxonate.

S-1 is widely administered to gastric cancer patients and consists of tegafur (FT), gimeracil (CDHP) and potassium oxonate (Oxo; 1,2,3,4-etetrahydro-2,4-dioxo-1,3,5-triazin-6-carboxylate) in a molar ratio of 1:0.4:1 (1–7). FT is converted to 5-fluorouracil (5-FU), which has an anticancer role *in vivo*. However, 5-FU has some disadvantages, including a short half-life *in vivo*, which causes gastrointestinal tract and bone marrow toxicity. CDHP and Oxo act as modulators, which have no anticancer activity when used singly. CDHP competitively inhibits dihydropyrimidine dehydrogenase to maintain a high plasma concentration of 5-FU (8). Oxo relieves the gastrointestinal tract toxicity induced by 5-FU (9). CDHP and Oxo facilitate the function of 5-FU, which leads to improved therapeutic effects (10). Oxo is converted into oxonic acid (Fig. 1) *in vivo*.

To date, there are several methods reported in the literature to determine Oxo, including enzyme immunoassay (11), GC-NICI-MS (gas chromatography-negative ion chemical ionization mass spectrometry) (12,13) and LC-MS/MS (liquid chromatography-tandem mass spectrometry) (14–17). The enzyme immunoassay method is not suitable for analysis in large batches. The GC-NICI-MS method has been shown to be unstable when used clinically. The LC-MS/MS method is reported to require long and complex pre-processing for derivatization. Therefore, a simple and novel method is required. In this study, a novel HPLC-MS/MS method was developed and successfully applied to a pharmacokinetic study, after single oral administration of a combination of FT, CDHP and Oxo to 12 tumor patients. This method was determined to be simple, rapid and stable for use in the quantitation of Oxo in human plasma.

## Materials and methods

### Chemicals and reagents

Potassium oxonate (purity, 99.60%) was supplied by the National Institute for the Control of Pharmaceutical and Biological Products (Beijing, China). [^13^C_2_,^15^N_3_]-Oxo (purity, 99.8%) was purchased from Toronto Research Chemicals Inc. (Toronto, ON, Canada). Methanol, acetonitrile and formic acid (LC grade) were purchased from Dima Technology Inc. (Richmond Hill, ON, Canada). The other chemicals (analytical grade) were supplied by Beijing Chemical Co. (Beijing, China). Deionized H_2_O was prepared using a Milli-Q H_2_O purifying system, purchased from Millipore Corporation (Bedford, MA, USA). Blank (drug-free) human plasma was obtained from healthy subjects.

### Preparation of stock and working solutions

The stock solutions were prepared separately in methanol-H_2_O [1:1, v/v; 400.0 *μ*g/ml for Oxo and 40.0 *μ*g/ml for the internal standard (IS)]. Routine daily calibration curves were prepared in drug-free plasma. Appropriate volumes of stock solutions and drug-free human plasma were added to each test tube to prepare working solutions at concentrations of 2.0, 5.0, 10.0, 25.0, 50.0, 100.0 and 200.0 ng/ml. Contemporary quality control samples, which were run in each assay, were also prepared at concentrations of 5.0, 25.0 and 160.0 ng/ml. Working solutions of IS (250.0 ng/ml) were obtained by diluting the stock solution with methano-H_2_O (1:1, v/v). The standard solutions and IS were stored at −20°C.

### HPLC-MS/MS analysis

HPLC was performed on a Shimadzu HPLC system consisting of a LC-20AD binary pump, a DGU-20A3 degasser, a SIL-20A autosampler and a CTO-10AS*_VP_* column oven (Shimadzu Corporation, Kyoto, Japan). Chromatographic separation was achieved on a Waters:Atlantis dC_18_ column (150x4.6 mm, 5.0 *μ*m, Waters, Milford, MA, USA) maintained at 20°C in the column oven. The signal acquisition and peak integration were performed by Analyst 1.4.2 software (Applied Biosystems, Foster City, CA, USA). The mobile phase was H_2_O containing 0.1% formic acid-acetonitrile (90:10, v/v), with a flow rate of 1.2 ml/min (70% shunting). Detection was carried out on an API 3200 MS/MS System (Applied Biosystems) equipped with an electrospray ionization (ESI) source in the negative ionization mode. Multiple reaction monitoring (MRM) at unit resolution was employed to monitor the transitions of Oxo at m/z 156.0→111.9 and IS at m/z 161.1→117.1. The operating parameters of ESI-mass spectrometry (ESI-MS) were as follows: curtain gas, 15 psi; gas 1, 40 psi; gas 2 (nitrogen), 80 psi; dwell time, 150 msec; ion spray voltage, −4.5 kV; ion source temperature, 550°C; declustering potential (DP), −20 V; collision energy (CE), −11 V.

### Sample preparation

Briefly, 50 *μ*l IS working solution and 200 *μ*l deionized H_2_O were added to a 50-*μ*l aliquot of human plasma in a 1.5-ml Eppendorf tube. The mixture was vortex-mixed for 30 sec at 20°C. The SPE column was activated by the elution of 350 *μ*l methanol and 350 *μ*l H_2_O. Plasma samples were loaded to the SPE column and the column was eluted by 350 *μ*l H_2_O and 350 *μ*l methanol. Subsequently, 25 *μ*l of the mixture of methanol and formic acid (50:50, v/v) was applied to extract Oxo twice. The eluted solution was vortex-mixed with 50 *μ*l H_2_O. Finally, 5 *μ*l of the previously mentioned solutions was injected and analyzed using HPLC-MS/MS.

### Pharmacokinetic study

Plasma concentrations of Oxo in 12 tumor patients were determined up to 48 h following single oral administration of a 40-mg S-1 capsule. Blood samples were drawn at various time-points (0.25, 0.5, 1, 1.5, 2, 3, 4, 6, 8, 10, 12, 24 and 48 h) after ingestion of S-1. This clinical pharmacokinetic study was approved by the Ethics Committee of the Affiliated Hospital of the Academy of Military Medical Sciences. All tumor patients provided written informed consent to participate in the study, in accordance with the principles of the Declaration of Helsinki.

## Results and Discussion

### Selection of IS

It is necessary to use an IS to obtain high accuracy when a mass spectrometer is used as the HPLC detector. [^13^C_2_,^15^N_3_]-Oxo was adopted as the IS due to the similarities in its retention and ionization characteristics with those of Oxo and the minimal endogenous interferences of [^13^C_2_,^15^N_3_]-Oxo in the MRM channels.

### Chromatography

**Figure 2.**
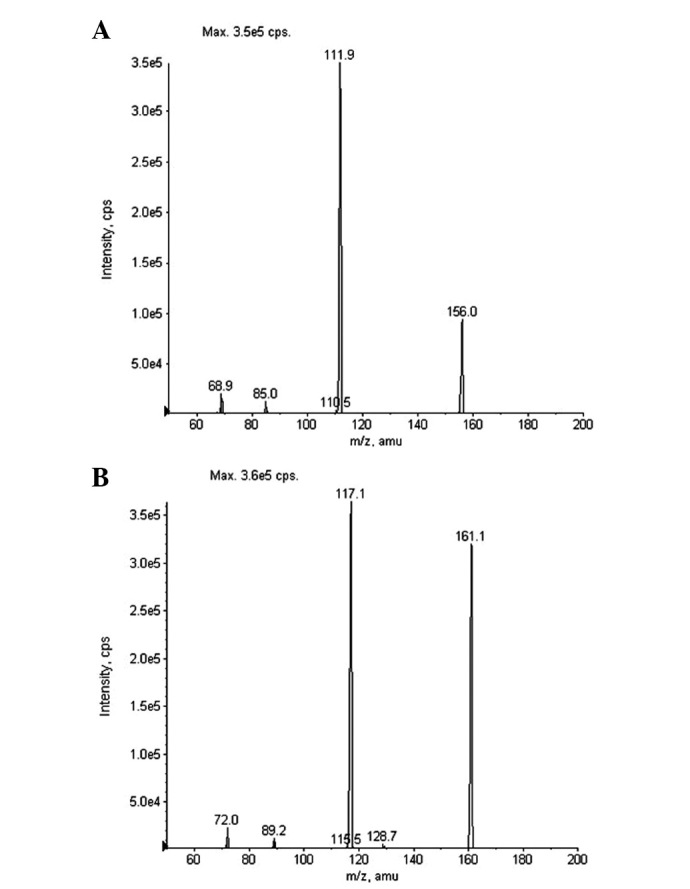
Full-scan product ion scans of [M–H]^−^ ions for (A) Oxo and (B) [^13^C_2_,^15^N_3_]-Oxo (IS). Oxo, potassium oxonate.

**Figure 3.**
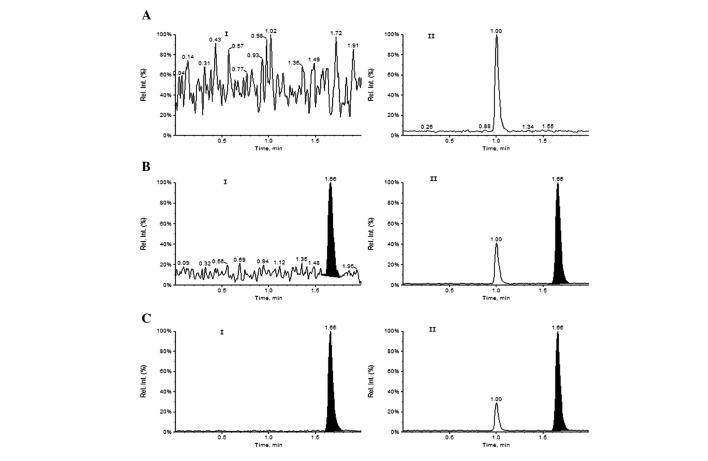
Typical MRM chromatograms of (I) Oxo and (II) IS obtained from human plasma samples. (A) Blank plasma; (B) blank plasma spiked with standard solution (LOQ); (C) plasma sample from a tumor patient 1.5 h after single oral administration of a 40-mg S-1 capsule with a concentration of 26.6 ng/ml. Oxo, potassium oxonate; IS, internal standard; LOQ, lower limit of quantification.

The stock solutions of Oxo and IS were diluted to a concentration of 1 *μ*g/ml with methanol. The diluted solutions (5 *μ*l) were injected and analyzed by the HPLC-MS/MS method. The mass spectra fragmentation of the ions from Oxo and IS are shown in Fig. 2. Molecular ions of Oxo and IS exhibited m/z 156.0 and 161.1 ([M–H]^−^), respectively. The product ion scan spectra revealed high abundance fragment ions at m/z 111.9 and 117.1 ([M–H]^−^) for Oxo and IS, respectively. Therefore, MRM transitions of m/z 156.0→111.9 and 161.1→117.1 were adopted for quantification of Oxo and IS, respectively. The typical HPLC-MS/MS MRM chromatograms of a blank human plasma sample and a human plasma sample spiked with Oxo and IS at the lower limit of quantification (LOQ; 2.0 ng/ml) are shown in Fig. 3. There was almost no interference peak at the retention time of Oxo (1.66 min) and IS (1.66 min). This result demonstrated that this method was sensitive and specific, allowing for the analysis of samples in batches and exhibiting suitability for pharmacokinetic studies.

### Linearity and LOQ

The calibration standards of seven Oxo concentration levels at 2.0, 5.0, 10.0, 25.0, 50.0, 100.0 and 200.0 ng/ml were injected and assayed. The calibration curve was constructed by plotting the peak area ratios (Y) of Oxo to the IS versus the concentrations (X) of Oxo, using weighted least squares linear regression (the weighting factor was 1/X^2^). In our study, the mean standard curve for Oxo was Y=0.0057X+0.000353 (r=0.9991). The concentrations of Oxo in unknown samples were obtained from the regression line. The LOQ was defined as the lowest concentration on the calibration curve, where precision was within ±20% and accuracy was within ±20%. This was established using six independent samples of standards.

### Intra-day and inter-day precision and accuracy

Intra-assay precision and accuracy were assessed by measuring the concentration of Oxo in six aliquots of three different quality control samples, which were extracted and analyzed on the same day. Inter-assay precision and accuracy were determined from the results of three different quality control samples, which were extracted and analyzed six-fold on three consecutive days. The results are presented in Table I.

### Matrix effect (ME)

The ME represents the potential ion suppression or enhancement effects of co-eluting and undetected matrix components in plasma. This was obtained by comparing the peak area of Oxo and IS spiked into post-extracted blank plasma samples to that of Oxo and IS spiked into the mobile phase at an equivalent concentration. In this study, the ME was evaluated by three quality control concentrations (5.0, 25.0 and 160.0 ng/ml) of Oxo and an IS concentration level of 250.0 ng/ml. Six samples at each concentration level were analyzed. The blank plasma used in this study was obtained from six different batches. If the peak area ratios were <85 or >115%, an endogenous ME was implied. The ME of plasma at concentrations of 5.0, 25.0 and 160.0 ng/ml were 95.50, 100.45 and 97.61%, respectively. The ME of IS was 95.6%. The results obtained were within the acceptable limit, suggesting that there was no ME observed in this study.

### Stability

The stability of Oxo in plasma under various conditions was evaluated. The quality control plasma samples (5.0, 25.0 and 160.0 ng/ml) were stable when placed at room temperature for 4 h, following three freeze/thaw (−40°C) cycles and when stored at −40°C for 3 months. The processed sample, placed in the autosampler at an ambient temperature (20°C) for 2 h, was also stable. These results (Table II) demonstrated that no significant degradation occurred under different conditions.

### Pharmacokinetic application

**Figure 4.**
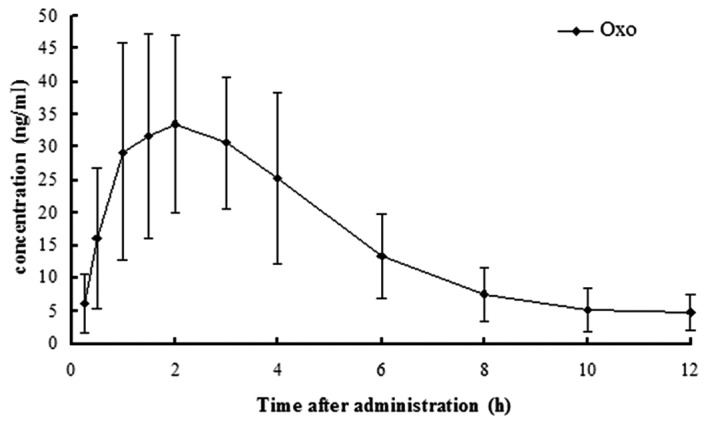
Mean plasma concentration-time profile in 12 tumor patients following single oral administration of a 40-mg S-1 capsule.

The method was applied for the analysis of plasma samples obtained from 12 tumor patients, following single oral administration of a 40-mg S-1 capsule in the pharmacokinetics study. The pharmacokinetic parameters were estimated by DAS version 2.1.1 software. Pharmacokinetic analysis of Oxo was performed using the noncompartmental method. The concentration maximum (C_max_) and the time to reach it (T_max_) were recorded directly. The elimination rate constant (K_e_) was calculated using linear regression of the terminal points from the semi-log plot of plasma concentration against time. The elimination half-life (t_1/2_) was calculated as 0.693/K_e_. The area under the plasma concentration-time curve of Oxo, from time zero to infinity (AUC_0-∞_), was determined using the linear trapezoidal rule to the last measurable plasma concentration (C_t_), plus the additional area from time t to infinity, calculated as C_t_/K_e_. Finally, the mean plasma concentration versus time profile and pharmacokinetic parameters of Oxo were obtained (Fig. 4; Table III). The primary pharmacokinetic parameters of Oxo in our study were similar to those as previously published (18–20). The method developed in this study was demonstrated to be accurate and sensitive when compared with the pharmacokinetic parameters and concentration-time curves of previous studies.

### Conclusions

A HPLC-MS/MS method was developed for the determination of Oxo in human plasma. There were numerous advantages to this method, including low volumes of sample requirement, simple sample processing, absence of the ME and a short analysis time. The method was successfully applied to a pharmacokinetic study after single oral administration of a 40-mg S-1 capsule in 12 tumor patients.

## Figures and Tables

**Table I.** Extraction recoveries, intra- and inter-day accuracy and precision.

	Extraction recoveries (n=5)		Accuracy (%, n=6)		Intra-day precision (n=6)		Inter-day precision (n=6)	
Concentration (ng/ml)	Recoveries (%)	RSD (%)	Intra-day	Inter-day	Mean	RSD (%)	Mean	RSD (%)
5	60.26	4.09	102.20	101.60	5.11	5.01	5.08	6.33
25	67.13	1.48	100.12	102.52	25.03	1.49	25.63	3.19
160	64.37	2.21	99.38	102.71	159.00	3.35	164.33	3.63

Extraction recovery (%) = [Mean peak area (extraction samples)/mean peak area (reference samples)] × 100. RSD, relative standard deviation.

**Table II.** Stability test for Oxo (n=5).

	Room temperature		−40°C for 3 months		Freeze/thaw		Autosampler	
Concentration (ng/ml)	Mean	RSD (%)	Mean	RSD (%)	Mean	RSD (%)	Mean	RSD (%)
5	5.16	7.40	4.69	8.90	5.27	3.66	4.83	5.92
25	25.77	2.94	25.03	4.83	25.97	3.47	23.83	2.42
160	166.00	4.93	163.67	3.14	163.00	1.62	158.33	2.85

Oxo, potassium oxonate; RSD, relative standard deviation.

**Table III.** Pharmacokinetic parameters of Oxo after single oral administration of a 40-mg S-1 capsule in 12 tumor patients.

Value	C_max_ (ng/ml)	T_max_ (h)	T_1/2_ (h)	AUC_(0–12)_ (ng/h/ml)	AUC_(0-∞)_ (ng/h/ml)
Mean	39.56	2.29	2.78	184.96	202.61
SD	14.37	0.89	0.99	70.12	85.03

Oxo, potassium oxonate; C_max_, peak plasma concentration; T_max_, time to reach C_max_; T_1/2_, elimination half-life; AUC_(0–12)_, area under the curve from 0 to 12 h; AUC_(0-∞)_, area under the curve from 0 to infinity; SD, standard deviation.

## References

[1] Kubota T (2008). The role of S-1 in the treatment of gastric cancer. Br J Cancer.

[2] Shirasaka T, Nakano K, Takechi T (1996). Antitumor activity of 1 M tegafur-0.4 M 5-chloro-2,4-dihydroxypyridine-1 M potassium oxonate (S-1) against human colon carcinoma ortho-topically implanted into nude rats. Cancer Res.

[3] Blum M, Suzuki A, Ajani JA (2011). A comprehensive review of S-1 in the treatment of advanced gastric adenocarcinoma. Future Oncol.

[4] Shimoyama S, Kiyokawa T, Nishida M, Seto Y (2012). S-1 monotherapy achieved twenty-month survival for peritoneal lavage cytology-positive gastric cancer patient undergoing noncurative resection. Gan To Kagaku Ryoho.

[5] Yamashita T, Arai K, Terashima T (2012). A case report of S-1 monotherapy for advanced hepatocellular carcinoma. Gan To Kagaku Ryoho.

[6] Iwasa S, Yamada Y, Kato K, Goto A, Honma Y, Hamaguchi T, Shimada Y (2012). Long-term results of a phase II study of S-1 plus irinotecan in metastatic colorectal cancer. Anticancer Res.

[7] Kogashiwa Y, Nagafuji H, Kohno N (2012). Feasibility of concurrent chemoradiotherapy with S-1 administered on alternate days for elderly patients with head and neck cancer. Anticancer Res.

[8] Shirasaka T, Shimamato Y, Ohshimo H (1996). Development of a novel form of an oral 5-fluorouracil derivative (S-1) directed to the potentiation of the tumor selective cytotoxicity of 5-fluorouracil by two biochemical modulators. Anticancer Drugs.

[9] Shirasaka T, Shimamoto Y, Fukushima M (1993). Inhibition by oxonic acid of gastrointestinal toxicity of 5-fluorouracil without loss of its antitumor activity in rats. Cancer Res.

[10] American Association for Cancer Research (1995).

[11] Kitamura R, Satoh T, Maeda M, Tsuji A (1994). Enzyme immunoassay of potassium oxonate using specific antibody isolated by immunosorbent gel. Yakugaku Zasshi.

[12] Matsushima E, Yoshida K, Kitamura R, Yoshida K (1997). Determination of S-1 (combined drug of tegafur, 5-chloro-2,4-dihydroxypyridine and potassium oxonate) and 5-fluorouracil in human plasma and urine using high-performance liquid chromatography and gas chromatography-negative ion chemical ionization mass spectrometry. J Chromatogr B Biomed Sci Appl.

[13] Schwaninger AE, Meyer MR, Huestis MA, Maurer HH (2011). Development and validation of LC-HRMS and GC-NICI-MS methods for stereoselective determination of MDMA and its phase I and II metabolites in human urine. J Mass Spectrom.

[14] Liu K, Zhong D, Zou H, Chen X (2010). Determination of tegafur, 5-fluorouracil, gimeracil and oxonic acid in human plasma using liquid chromatography-tandem mass spectrometry. J Pharm Biomed Anal.

[15] Star-Weinstock M, Williamson BL, Dey S, Pillai S, Purkayastha S (2012). LC-ESI-MS/MS Analysis of Testosterone at Sub-Picogram Levels Using a Novel Derivatization Reagent. Anal Chem.

[16] Giorgianni F, Mileo V, Desiderio DM, Catinella S, Beranova-Giorgianni S (2012). Characterization of the phosphoproteome in human bronchoalveolar lavage fluid. Int J Proteomics.

[17] de Mateo S, Estanyol JM, Oliva R (2013). Methods for the analysis of the sperm proteome. Methods Mol Biol.

[18] Hirata K, Horikoshi N, Aiba K (1999). Pharmacokinetic study of S-1, a novel oral fluorouracil antitumor drug. Clin Cancer Res.

[19] Peters GJ, Noordhuis P, Van Kuilenburg AB (2003). Pharmacokinetics of S-1, an oral formulation of ftorafur, oxonic acid and 5-chloro-2,4-dihydroxypyridine (molar ratio 1:0.4:1) in patients with solid tumors. Cancer Chemother Pharmacol.

[20] Mende B, Krauss J, Thyssen D (2009). Pharmacokinetic study of S-1. Int J Clin Pharmacol Ther.

